# Concentration-Dependent Binding of Small Ligands to Multiple Saturable Sites in Membrane Proteins

**DOI:** 10.1038/s41598-017-05896-8

**Published:** 2017-07-18

**Authors:** Letícia Stock, Juliana Hosoume, Werner Treptow

**Affiliations:** Laboratório de Biologia Teórica e Computacional (LBTC), Universidade de Brasília DF, Brasília, DF Brazil

## Abstract

Membrane proteins are primary targets for most therapeutic indications in cancer and neurological diseases, binding over 50% of all known small molecule drugs. Understanding how such ligands impact membrane proteins requires knowledge on the molecular structure of ligand binding, a reasoning that has driven relentless efforts in drug discovery and translational research. Binding of small ligands appears however highly complex involving interaction to multiple transmembrane protein sites featuring single or multiple occupancy states. Within this scenario, looking for new developments in the field, we investigate the concentration-dependent binding of ligands to multiple saturable sites in membrane proteins. The study relying on docking and free-energy perturbation provides us with an extensive description of the probability density of protein-ligand states that allows for computation of thermodynamic properties of interest. It also provides one- and three-dimensional spatial descriptions for the ligand density across the protein-membrane system which can be of interest for structural purposes. Illustration and discussion of the results are shown for binding of the general anesthetic sevoflurane against Kv1.2, a mammalian ion channel for which experimental data are available.

## Introduction

Membrane proteins are critical for diverse processes in cells. Given their relevance, membrane proteins are targets for a large family of ligands, including small drug molecules featuring a wide spectrum of pharmaceutical properties. How such ligands modulate the function of membrane proteins must at some point build on understanding ligand binding, a reasoning that has driven growing efforts in the field^1^. Currently, though not refuting a membrane-mediated mechanism in which ligands may impact proteins indirectly through modification of bilayer properties^2^, high-resolution measurements and manifold studies involving molecular dynamics (MD) support that small ligands bind membrane proteins at low concentrations^3–14^. Specifically, partitioning of such molecules across the water-membrane phases allows accessibility-to and binding-to multiple transmembrane (TM) protein sites featuring single or multiple occupancy states. In contrast to typical one-to-one substrate interactions against enzymes, binding of small ligands to membrane receptors appears highly complex and might depend further on chemotypes, protein-types and conformations as recently evidenced in structural studies of ion channels^5, 11^. Within this scenario, solution of the problem implies demonstrating ligand binding and determining from first principles how that affects protein equilibrium to modulate function. Although some progress has been made in one or more of these aspects, a detailed description of the problem still misses.

Here, as a pre-requisite to solve this hierarchical problem, we investigate ligand binding to a specific protein conformation that features multiple sites occupied by one or more ligands in a concentration-dependent manner. For that purpose, we present a rigorous theoretical framework to tackle the problem via a combined docking and free-energy perturbation (FEP) approach. In part, the framework represents an extension of previous treatment dealing with binding of water molecules to a single receptor site of the bacteriorhodopsin proton channel^15^. Specifically, the theory provides us with a complete description of the probability density of the protein-ligand bound states allowing for computation of any thermodynamic properties of interest. Besides that, a three-dimensional description of the binding problem is readily derived by mapping state-dependent into space-dependent probability densities of the ligand which can be of special interest for structural measurements. Illustration and discussion of the approach is presented for binding of the general anesthetic sevoflurane against Kv1.2, a mammalian voltage-gated potassium channel for which experimental data are available.

## Theory and Methods

Consider a microscopic system constituted by $M$ molecular species in thermodynamic equilibrium. The potential energy of the system is $U({{\boldsymbol{r}}}^{M})$ with ${{\boldsymbol{r}}}^{M}\equiv \{{{\boldsymbol{r}}}_{1},\ldots ,{{\boldsymbol{r}}}_{N},{{\boldsymbol{r}}}_{N+1},\ldots ,{{\boldsymbol{r}}}_{M}\}$ denoting the degrees of freedom of the *M* molecular constituents; ***r***_*i*_ is a shorthand for the entire set of Cartesian coordinates of molecule *i*. The system comprises of a single protein receptor fixed at the origin of the coordinate system and embedded in a *large* membrane-aqueous volume that contains *N* identical ligands under dilute conditions. The protein is assumed to remain in a well-defined conformational state providing with *s* distinct binding sites for ligands. For simplicity, we consider that ligands dissolve uniformly across the membrane-aqueous region of the system from where they can partition into the protein binding sites. The lipid and aqueous phases thus provide with a reservoir volume *V* occupied by ligands at constant density $\bar{{\rm{\rho }}}$ and *excess* chemical potential $\bar{{\rm{\mu }}}$. We consider further that every site $j=1,\ldots ,s$ corresponds to a discrete volume δ*V*_*j*_ that can be populated by $0\leqslant {n}_{j}\leqslant {n}_{j}^{max}$ ligands. Then, there is a maximum number of bound *O* states accessible to the protein receptor in the system, $max(O)={\prod }_{j=1}^{s}({n}_{j}^{max}+1)$. We denote by $O({n}_{1},\ldots ,{n}_{s})$ the specific state featuring exactly *n*_*j*_ bound ligands at corresponding sites and by $n={n}_{1}+\ldots +{n}_{s}$ the total number of bound ligands in this state.

### Equilibrium binding constant

Under these considerations, solution of ligand binding to multiple receptor sites relies fundamentally on determining the equilibrium constant ${\rm{{\rm K}}}({n}_{1},\ldots ,{n}_{s})$ for the process $O({0}_{1},\ldots ,{0}_{s})+nL\iff O({n}_{1},\ldots ,{n}_{s})$ where, $O({0}_{1},\ldots ,{0}_{s})$ is the empty receptor state with all ligands occupying the system reservoir^16–19^. Because the system is dilute, ${\rm{{\rm K}}}({n}_{1},\ldots ,{n}_{s})$ relates to the microscopic probability densities of each of the reaction species at equilibrium

1 $${\rm{{\rm K}}}({n}_{1},\ldots ,{n}_{s})=\frac{1}{{\bar{{\rm{\rho }}}}^{n}}\times \frac{{\rm{\rho }}({n}_{1},\ldots ,{n}_{s})}{{\rm{\rho }}({0}_{1},\ldots ,{0}_{s})}$$

in which, $\rho ({n}_{1},\ldots ,{n}_{s})$ denotes the probability of finding the protein receptor at the ligand-bound state *O*(*n*_1_, …, *n*_*s*_). Note that for dilute solutions, equation (1) is equivalent to its classical definition in terms of the concentration of each of the species in the process.

For a fixed temperature ${\rm{\beta }}={({k}_{B}T)}^{-1}$, $\rho ({n}_{1},\ldots ,{n}_{s})$ is formally expressed as the canonical probability density of the system^20^ which allows restatement of equation (1)

2 $${\rm{K}}({n}_{1},\ldots ,{n}_{s})=\frac{1}{{\bar{{\rm{\rho }}}}^{n}}\times \tfrac{N!}{{n}_{1}!\ldots {n}_{s}!(N-n)!}\tfrac{{\int }_{{\rm{\delta }}{V}_{1}}d\,{{\boldsymbol{r}}}^{{n}_{1}}\ldots {\int }_{{\rm{\delta }}{V}_{s}}d\,{{\boldsymbol{r}}}^{{n}_{s}}{\int }_{V}d\,{{\boldsymbol{r}}}^{N-n}\int d\,{{\boldsymbol{r}}}^{M-N}{e}^{-{\rm{\beta }}U({{\boldsymbol{r}}}^{M})}}{{\int }_{V}d\,{{\boldsymbol{r}}}^{N}\int d\,{{\boldsymbol{r}}}^{M-N}{e}^{-{\rm{\beta }}U({{\boldsymbol{r}}}^{M})}}$$

in terms of configuration integrals over the ligand-bound $O({n}_{1},\ldots ,{n}_{s})$ and ligand-free $O({0}_{1},\ldots ,{0}_{s})$ states of the protein. Here, the volume integral over the reservoir and site specific regions of the system are restricted to microscopic configurations ***r*** ^*M*^ accessible to $O({n}_{1},\ldots ,{n}_{s})$ and $\frac{N!}{{n}_{1}!\ldots {n}_{s}!(N-n)!}$ corrects for the degeneracy of the state given the indistinguishable nature of the ligands.

In the present form, equation (2) can be evaluated in the context of MD simulations and free-energy perturbation (FEP) calculations^21^ by taking into consideration the reversible work *W*(***R*** ^*n*^) associated with the centroid configuration ${{\boldsymbol{R}}}^{n}\equiv \{{{\boldsymbol{R}}}_{1},\ldots ,{{\boldsymbol{R}}}_{n}\}$ of *n* ligands

3 $${\rm{K}}({n}_{1},\ldots ,{n}_{s})=\frac{1}{{\bar{{\rm{\rho }}}}^{n}}\times \tfrac{N!}{{n}_{1}!\ldots {n}_{s}!(N-n)!}\tfrac{{\int }_{{\rm{\delta }}{V}_{1}}d\,{{\boldsymbol{R}}}^{{n}_{1}}\ldots {\int }_{{\rm{\delta }}{V}_{s}}d\,{{\boldsymbol{R}}}^{{n}_{s}}{e}^{-{\rm{\beta }}W({{\boldsymbol{R}}}^{n})}}{{\int }_{V}d\,{{\boldsymbol{R}}}^{n}{e}^{-{\rm{\beta }}W({{\boldsymbol{R}}}^{n})}}$$

where,

$${e}^{-\beta W({{\boldsymbol{R}}}^{n})}=\frac{\int d{{\boldsymbol{r}}}_{1}\delta [{{\boldsymbol{R}}}_{1}^{^{\prime} }({{\boldsymbol{r}}}_{1})-{{\boldsymbol{R}}}_{1}]\ldots \int d{{\boldsymbol{r}}}_{n}\delta [{{\boldsymbol{R}}}_{n}^{^{\prime} }({{\boldsymbol{r}}}_{n})-{{\boldsymbol{R}}}_{n}]\int d{{\boldsymbol{r}}}^{N-n}\int d{{\boldsymbol{r}}}^{M-N}{e}^{-\beta U({{\boldsymbol{r}}}^{M})}}{\int d{{\boldsymbol{r}}}_{1}\delta [{{\boldsymbol{R}}^{\prime} }_{1}({{\boldsymbol{r}}}_{1})-{{\boldsymbol{R}}}_{1}^{\ast }]\ldots \int d{{\boldsymbol{r}}}_{n}\delta [{{\boldsymbol{R}}^{\prime} }_{n}({{\boldsymbol{r}}}_{n})-{{\boldsymbol{R}}}_{n}^{\ast }]\int d{{\boldsymbol{r}}}^{N-n}\int d{{\boldsymbol{r}}}^{M-N}{e}^{-\beta {U}_{o}({{\boldsymbol{r}}}^{M})}}$$

is defined relative to a reference system ${U}_{o}({{\boldsymbol{r}}}^{M})$ in which all the interactions of the *n* ligands with the remaining particles of the system are switched off. Here, ${{\boldsymbol{R}}}_{i}({{\boldsymbol{r}}}_{i})$ is explicitly given

$${{\boldsymbol{R}}}_{i}({{\boldsymbol{r}}}_{i})\equiv \frac{1}{{N}_{i}^{atom}}\sum _{(x,y,z)\in {{\boldsymbol{r}}}_{i}}(x,y,z)$$

from the Cartesian coordinates of the ${N}_{i}^{atom}$ atoms of ligand *i* and $\{{{\boldsymbol{R}}}_{1}^{\ast },\ldots ,{{\boldsymbol{R}}}_{n}^{\ast }\}$ is any arbitrary set of reference positions of the ligands in the system. Within this definition, *W*(***R*** ^*n*^) corresponds to the free energy variation associated with transfer of ligands from gas phase to their molecular environments. Note that by construction, the single-molecule reversible work *W*(***R***_*i*_) has a simple connection with the *excess* chemical potential $\bar{{\rm{\mu }}}$ (or identically, the solvation free energy) for any position ***R***_*i*_ of the ligand in the reservoir that is, ${e}^{-\beta W({{\boldsymbol{R}}}_{i})}={e}^{-\beta \bar{{\rm{\mu }}}}$. The implications for the reservoir integral of *n* ligands in equation (3) are then clear

4 $${\int }_{V}d\,{{\boldsymbol{R}}}^{n}{e}^{-{\rm{\beta }}W({{\boldsymbol{R}}}^{n})}={V}^{n}{e}^{-{\rm{\beta }}n\bar{{\rm{\mu }}}}$$

given their weak couplings in the reservoir volume under dilute conditions that is, $W({{\boldsymbol{R}}}^{n})\approx W({{\boldsymbol{R}}}_{1})+$ $\ldots +W({{\boldsymbol{R}}}_{n})$. As presented in Computational Methods, ${e}^{-{\rm{\beta }}\bar{{\rm{\mu }}}}$ can be evaluated from one single FEP calculation handling the reversible decoupling of the ligand from the reservoir. On the other hand, estimation of the site specific integral in equation (3) by means of FEP requires the use of an auxiliary external potential to ensure an accurate sampling of the ligand in the binding site volume. This is critical to ensure that the ligand has a well defined chemical potential at the last stages of decoupling from the protein cavity^15, 16^. By defining a harmonic potential coupled to ligand *i*

$${u}^{\ast }({{\boldsymbol{R}}}_{i})=\frac{1}{2}{k}_{j}{[{{\boldsymbol{R}}}_{i}-{{\boldsymbol{R}}}_{i}^{\ast }]}^{2}$$

in terms of a reference position ${{\boldsymbol{R}}}_{i}^{\ast }$ of the ligand at the binding site then

5 $${\int }_{{\rm{\delta }}{V}_{1}}d\,{{\boldsymbol{R}}}^{{n}_{1}}\ldots {\int }_{{\rm{\delta }}{V}_{s}}d\,{{\boldsymbol{R}}}^{{n}_{s}}{e}^{-{\rm{\beta }}W({{\boldsymbol{R}}}^{n})}=[\prod _{i=1}^{n}{(\frac{2\pi }{{\rm{\beta }}{k}_{i}})}^{\frac{3}{2}}]{e}^{-{\rm{\beta }}{W}_{n}^{\ast }}$$

in which ${W}_{n}^{\ast }$ corresponds to the free-energy of *n* site-specific bound ligands relative to a gas phase state given that the same ligands $i=1,\ldots ,n$ are restrained to occupy an *effective* volume $[{\prod }_{i=1}^{n}{(\frac{2\pi }{\beta {k}_{i}})}^{\frac{3}{2}}]$ centered at the equilibrium positions ${{\boldsymbol{R}}}_{i}^{\ast }$ at the protein sites (*cf*. Supplementary Information text for details). Equations (3), (4) and (5) thus establish that

6 $$K({n}_{1},\ldots ,{n}_{s})=\frac{1}{{n}_{1}!\ldots {n}_{s}!}[\prod _{i=1}^{n}{(\frac{2\pi }{{\rm{\beta }}{k}_{i}})}^{\frac{3}{2}}]{e}^{-{\rm{\beta }}[{W}_{n}^{\ast }-n\bar{{\rm{\mu }}}]}$$

for the thermodynamic limit $N\gg n$, $\frac{N!}{(N-n)!}\approx {N}^{n}$. Here, $\frac{1}{{n}_{1}!\ldots {n}_{s}!}$ corrects the equilibrium constant for the equivalent configurations of *n*_*j*_ indistinguishable ligands within the site volumes δ*V*_*j*_.

By describing the equilibrium constant in terms of ligand binding over multiple protein sites, equations (1) to (6) are generalizations of the formulation in reference^15^ dealing with water occupancy of the bacteriorhodopsin proton channel. Within this formulation, knowledge of ${\rm{{\rm K}}}({n}_{1},\ldots ,{n}_{s})$ ensures the probability of any occupancy state

7 $${\rm{\rho }}({n}_{1},\ldots ,{n}_{s})=\frac{{\bar{{\rm{\rho }}}}^{({n}_{1}+\ldots +{n}_{s})}\,K({n}_{1},\ldots ,{n}_{s})}{{\sum }_{{n}_{1}^{^{\prime} },\ldots ,{n}_{s}^{^{\prime} }}{\bar{{\rm{\rho }}}}^{({n}_{1}^{^{\prime} }+\ldots +{n}_{s}^{^{\prime} })}\,K({n}_{1}^{^{\prime} },\ldots ,{n}_{s}^{^{\prime} })}$$

to be known in principle from free-energy calculations. Here, the normalization condition appearing on the denominator of equation (7) runs from the occupancy state $O({0}_{1},\ldots ,{0}_{s})$ up to $O({n}_{1}^{max},\ldots ,{n}_{s}^{max})$. The relevance of the result is clear

8 $$\langle A\rangle =\sum _{{n}_{1}^{^{\prime} },\ldots ,{n}_{s}^{^{\prime} }}{\langle A\rangle }_{({n}_{1}^{^{\prime} },\ldots ,{n}_{s}^{^{\prime} })}{\rm{\rho }}({n}_{1}^{^{\prime} },\ldots ,{n}_{s}^{^{\prime} })$$

as the ensemble average of any thermodynamic property of the system $A({n}_{1}^{^{\prime} },\ldots ,{n}_{s}^{^{\prime} })$ for state $O({n}_{1}^{\text{'}},\ldots ,{n}_{s}^{\text{'}})$ can be known from equation (8). Note, in equation (7), $\rho ({n}_{1},\ldots ,{n}_{s})$ depends on the density or concentration of the ligand in the reservoir thus providing us with a useful equation for investigation of concentration effects on binding.

From equation (6), an absolute binding free-energy ${\rm{\Delta }}G^\circ ({n}_{1},\ldots ,{n}_{s})$ ^16^ associated with state $O({n}_{1},\ldots ,{n}_{s})$ can be also defined as

9 $${\rm{\Delta }}G^\circ ({n}_{1},\ldots ,{n}_{s})=-{\beta }^{-1}\,\mathrm{ln}\,[K({n}_{1},\ldots ,{n}_{s})\times {(C^\circ )}^{n}]$$

where it is understood that this refers to the free energy of binding *n* ligands to the protein receptor from a reference standard reservoir concentration $C^\circ =1\,M$ or in units of number density $C^\circ ={(1,660{A}^{3})}^{-1}$. The standard free energy

$${\rm{\Delta }}G^\circ ({n}_{1},\ldots ,{n}_{s})=[{W}_{n}^{\ast }-n\bar{{\rm{\mu }}}]-{{\rm{\beta }}}^{-1}\,\mathrm{ln}\,[\prod _{i=1}^{n}C^\circ \times {(\frac{2{\rm{\pi }}}{{\rm{\beta }}{k}_{i}})}^{\frac{3}{2}}]-{{\rm{\beta }}}^{-1}\,\mathrm{ln}\,[\frac{1}{{n}_{1}!\ldots {n}_{s}!}]$$

then rewrites in terms of three contributions: *i*) the free-energy variation of binding from the gas phase under restrained potentials minus the *excess* potential, *ii*) the free-energy change when the restrained, gas-phase ligands are allowed to expand to occupy a volume ${(C^\circ )}^{-n}$ and *iii*) the free-energy correction for *n* indistinguishable ligands into the binding sites. Note that for the case of *n* = 1, this equation reduces to the familiar formulation considered in FEP studies, ${\rm{\Delta }}G^\circ (n=1)=[{W}_{1}^{\ast }-\bar{{\rm{\mu }}}]-{{\rm{\beta }}}^{-1}\,\mathrm{ln}\,[\frac{{V}_{effective}}{{V}_{o}}]$.

### Independent binding sites

Although rigorously correct and insightful, equation (6) cannot be applied to solve in practice multiple correlated binding events. The complexity in estimating ${W}_{n}^{\ast }$ from FEP increases significantly with the number of inter-correlated sites along the receptor structure. In contrast, equation (6) can be simplified under the condition of ligand interactions to multiple independent sites; a condition that we expect to be fulfilled in large membrane proteins featuring sparse binding sites for ligands. Within this scenario, the PMF *W*(*R* ^*n*^) for the bound state of *n* ligands can be approximated

10 $$W({{\boldsymbol{R}}}^{n})=W({{\boldsymbol{R}}}^{{n}_{1}},\ldots ,{{\boldsymbol{R}}}^{{n}_{s}})\approx W({{\boldsymbol{R}}}^{{n}_{1}})+\ldots +W({{\boldsymbol{R}}}^{{n}_{s}})$$

thus ensuring the binding constant ${\rm{{\rm K}}}({n}_{1},\ldots ,{n}_{s})$ to be factorized, as the product of independent equilibrium constants

11 $${\rm{{\rm K}}}({n}_{1},\ldots ,{n}_{s})={\rm{{\rm K}}}({n}_{1},{0}_{2}\ldots ,{0}_{s})\times \ldots \times {\rm{{\rm K}}}({0}_{1},\ldots ,{0}_{s-1},{n}_{s})$$

where,

12 $$\begin{array}{rcl}{\rm{K}}({n}_{1},{0}_{2},\ldots ,{n}_{s}) & = & \frac{1}{{n}_{1}!}[\prod _{i=1}^{{n}_{1}}{(\frac{2\pi }{{\rm{\beta }}{k}_{i}})}^{\frac{3}{2}}]{e}^{-{\rm{\beta }}[{W}_{{n}_{1}}^{\ast }-{n}_{1}\bar{{\rm{\mu }}}]}\\  &  & \cdots \\ {\rm{K}}({0}_{1},\ldots ,{0}_{s-1},{n}_{s}) & = & \frac{1}{{n}_{s}!}[\prod _{i=1}^{{n}_{s}}{(\frac{2\pi }{{\rm{\beta }}{k}_{i}})}^{\frac{3}{2}}]{e}^{-{\rm{\beta }}[{W}_{{n}_{s}}^{\ast }-{n}_{s}\bar{{\rm{\mu }}}]}\end{array}$$

denote respectively the binding constant of *n*_*j*_ ligands to each of the *j* sites on the receptor structure. Description of equation (11) is of practical relevance by allowing computation of ${\rm{{\rm K}}}({n}_{1},\ldots ,{n}_{s})$ from a series of independent and parallel free-energy calculations aimed at determining binding affinities individually.

### Position-dependent probability densities

So far, the treatment describes the probability density of states ${\rm{\rho }}({n}_{1},\ldots ,{n}_{s})$. For further progress, we explore mapping ${\rm{\rho }}({n}_{1},\ldots ,{n}_{s})$ into the probability density ρ(***R***) of any given ligand *i* to occupy position ***R*** in the system (regardless the position of the remaining *N* − 1 ligands). The probability density ρ(***R***) is given by

$${\rm{\rho }}({\boldsymbol{R}})=\frac{N}{\int d\,{{\boldsymbol{r}}}^{M}{e}^{-\beta U({r}^{M})}}\int d\,{{\boldsymbol{r}}}_{1}{\rm{\delta }}[{{\boldsymbol{R}}}_{1}^{^{\prime} }({{\boldsymbol{r}}}_{1})-{\boldsymbol{R}}]\ldots \int d\,{{\boldsymbol{r}}}_{N}\int d\,{{\boldsymbol{r}}}^{M-N}{e}^{-{\rm{\beta }}U({r}^{M})}$$

where, ***R***_1_(*r*_1_) is the instantaneous centroid position of ligand *i* = 1 in the system. The factor *N* accounts for equivalent configurations of the indistinguishable ligands. Given our original consideration that the reservoir is a homogeneous volume occupied by ligands with position-independent density $\bar{{\rm{\rho }}}$, the probability ρ(***R***) simplifies to

13 $${\rm{\rho }}({\boldsymbol{R}})=\{\begin{array}{l}{{\rm{\rho }}}_{j}({\boldsymbol{R}}),\,\forall \,{\boldsymbol{R}}\in {\rm{\delta }}{V}_{j}\\ \bar{{\rm{\rho }}},\,reservoir\end{array}$$

for every protein site $j=1,\ldots ,s$. The determination of ρ(***R***) thus reduces in practice to knowledge of ρ_*j*_(***R***) within each site.

By defining the configuration-dependent number of bound ligands ${n^{\prime} }_{j}({r}_{1},\ldots ,{r}_{N})$ as a function of their centroid positions ***R***_*i*_(***r***_*i*_) in the system

$${n}_{j}^{^{\prime} }({r}_{1},\ldots ,{r}_{N})\equiv \sum _{i=1}^{N}[\int d\,{\boldsymbol{R}}{\rm{\delta }}[{{\boldsymbol{R}}}_{i}({{\boldsymbol{r}}}_{i})-{\boldsymbol{R}}]]$$

the probability density ρ_*j*_(***R***)

$$\begin{array}{ccc}{\rho }_{j}({\boldsymbol{R}}) & = & {\textstyle \tfrac{N}{\int d{{\boldsymbol{r}}}^{M}{e}^{-\beta U({r}^{M})}}}\int d{{\boldsymbol{r}}}_{1}\delta [{{\boldsymbol{R}}}_{1}^{^{\prime} }({{\boldsymbol{r}}}_{1})-{\boldsymbol{R}}]\ldots \\  &  & \times \int d{{\boldsymbol{r}}}_{N}\mathop{\overbrace{[{\sum }_{{n}_{j}=0}^{{n}_{j}^{max}}\delta [{n}_{j}^{^{\prime} }({{\boldsymbol{r}}}_{1},\ldots ,{{\boldsymbol{r}}}_{N})-{n}_{j}]]}}\limits^{=1}\int d{{\boldsymbol{r}}}^{M-N}{e}^{-\beta U({{\boldsymbol{r}}}^{M})}\end{array}$$

can be restated in terms of discrete occupancy states of the binding site,

14 $$\begin{array}{rcl}{{\rm{\rho }}}_{j}({\boldsymbol{R}}) & = & \sum _{{n}_{j}=0}^{{n}_{j}^{max}}\frac{{\int }_{{n}_{j}^{^{\prime} }({{\boldsymbol{r}}}_{1},\ldots ,{{\boldsymbol{r}}}_{N})={n}_{j}}d\,{{\boldsymbol{r}}}_{1}\ldots {\int d{\boldsymbol{r}}}_{N}{\int d{\boldsymbol{r}}}^{M-N}{e}^{-\beta U({{\boldsymbol{r}}}^{M})}}{\int d\,{{\boldsymbol{r}}}_{1}\ldots \int d\,{{\boldsymbol{r}}}_{N}\int d\,{{\boldsymbol{r}}}^{M-N}{e}^{-\beta U({{\boldsymbol{r}}}^{M})}}\\  &  & \times \frac{N{\int }_{{n}_{j}^{^{\prime} }({{\boldsymbol{r}}}_{1},\ldots ,{{\boldsymbol{r}}}_{N})={n}_{j}}d\,{{\boldsymbol{r}}}_{1}{\rm{\delta }}[{{\boldsymbol{R}}}_{1}({{\boldsymbol{r}}}_{1})-{\boldsymbol{R}}]\ldots \int d\,{{\boldsymbol{r}}}_{N}\int d\,{{\boldsymbol{r}}}^{M-N}{e}^{-\beta U({{\boldsymbol{r}}}^{M})}}{{\int }_{{n}_{j}^{^{\prime} }({{\boldsymbol{r}}}_{1},\ldots ,{{\boldsymbol{r}}}_{N})={n}_{j}}d\,{{\boldsymbol{r}}}_{1}\ldots \int d\,{{\boldsymbol{r}}}_{N}\int d\,{{\boldsymbol{r}}}^{M-N}{e}^{-\beta U({{\boldsymbol{r}}}^{M})}}\\  & = & \sum _{{n}_{j}=0}^{{n}_{j}^{max}}{\rm{\rho }}({n}_{j})\times {\rm{\rho }}({\boldsymbol{R}}|{n}_{j})\end{array}$$

in which, ${\rm{\rho }}({\boldsymbol{R}}|{n}_{j})$ is the local density at site *j* when occupied exactly by *n*_*j*_ molecules and ρ(*n*_*j*_) is the probability for this occupancy state. In the definition of ${n^{\prime} }_{j}({r}_{1},\ldots ,{r}_{N})$, the Dirac delta functions ${\rm{\delta }}[{{\boldsymbol{R}}}_{i}({r}_{i})-{\boldsymbol{R}}]$ ensures counting of bound ligands within the site volume only. This definition is identical to that considered by Roux and coworkers^15^. In equation (14), ${\rm{\rho }}({\boldsymbol{R}}|{n}_{j})$ describes the local equilibrium density of the ligand, conditional to a specific number of bound molecules that satisfies ${\int }_{{\rm{\delta }}{V}_{j}}d\,{\boldsymbol{R}}{\rm{\rho }}({\boldsymbol{R}}|{n}_{j})={n}_{j}$. In contrast,

15 $${\rm{\rho }}({n}_{j})=\sum _{{n}_{1}^{^{\prime} },\ldots ,{n}_{s}^{^{\prime} }}{{\rm{\delta }}}_{{n}_{j}^{^{\prime} },{n}_{j}}{\rm{\rho }}({n}_{1}^{^{\prime} },\ldots ,{n}_{s}^{^{\prime} })$$

denotes the marginal probability of site *j* to be occupied by *n*_*j*_ ligands regardless the occupancy of the other sites.

Equations (13) and (14) establish a formal relation between space-dependent and state-dependent densities of the system. At a fine level, this relation involves the set of equilibrium constants ${\rm{{\rm K}}}({n}_{1},\ldots ,{n}_{s})$ satisfying ${\rm{\rho }}({n}_{j})$. The result can be of interest by embodying the probability densities of multiple occupancy states of the protein receptor into ρ(***R***). Besides that, equations (13) and (14) can be useful for analysis of spatial projections of ρ(***R***). For instance, the density profile along the *z* direction of the system can be achieved as

16 $${\rm{\rho }}(z)=\bar{{\rm{\rho }}}\times A(z)+\sum _{j=1}^{s}{{\rm{\rho }}}_{j}(z)$$

where, $A(z)={\rm{\Delta }}x{\rm{\Delta }}y$ is the total area of the membrane-aqueous region along the Cartesian *x* and *y* directions (*cf*. Supplementary Information for details).

### Symmetry of membrane proteins

For completeness, note that we have considered $j=1,\ldots ,s$ distinguishable sites for ligand binding on the protein receptor with volume δ*V*_*j*_ and site-specific affinity ${\rm{{\rm K}}}({0}_{1},\ldots ,{n}_{j},\ldots ,{0}_{s})$. Typically, membrane proteins are in average *f*-fold symmetric structures given their oligomeric nature. Therefore, there might be effectively $k=1,\ldots ,s^{\prime} \le s$ distinguishable sites for ligand binding across the protein subunits such that

$$\{\begin{array}{c}\delta {V}_{{\rm{j}}}=\delta {V}_{{\rm{k}}}\\ {\rm{K}}({0}_{1},\ldots ,{n}_{{\rm{j}}},\ldots ,{0}_{s})={\rm{K}}({0}_{1},\ldots ,{n}_{k},\ldots ,{0}_{s^{\prime} })\end{array}$$

for every $j\in {\{j\}}_{k}$. This implies that ${\rm{{\rm K}}}({n}_{1},\ldots ,{n}_{s})$ for any occupancy state of the protein involving *s* independent sites can be reconstructed in average

17 $${\rm{{\rm K}}}({n}_{1},\ldots ,{n}_{s})=\prod _{k=1}^{s^{\prime} }{\rm{{\rm K}}}{({0}_{1},\ldots ,{n}_{k},\ldots ,{0}_{s^{\prime} })}^{\chi ({n}_{k})}$$

from the individual affinities of *k* distinguishable sites allowing all previous results to be derived accordingly. Here, $\chi ({n}_{k})$ is the symmetry number for the site occupancy ${n}_{k}$ appearing in state $O({n}_{1},\ldots ,{n}_{s})$ - the symmetry number satisfies $s=\sum _{k=1}^{s\text{'}}\chi ({n}_{k})$.

The *f*-fold symmetry ensures ${\rm{{\rm K}}}({0}_{1},\ldots ,{n}_{k},\ldots ,{0}_{t})$ and derived results to be estimated from the average estimator

18 $${\bar{A}}_{k}=\frac{1}{f}\sum _{j\in {\{j\}}_{k}}{A}_{j}$$

over the set of indistinguishable independent sites $j\in {\{j\}}_{k}$, with associated statistical errors ${\sigma }_{\bar{A}}^{2}={\bar{A}}_{k}^{2}-{\bar{A}}_{k}^{2}$. Note that, ${\bar{A}}_{k}$ only converges ${\sigma }_{\bar{A}}^{2}\to 0$ in the limit of a complete ensemble for the protein conformation under consideration.

### Computational methods

A procedure was designed to solve the molecular binding of the haloether sevoflurane to the Kv1.2 channel under assumption of independent binding sites and saturation conditions up to ${n}_{j}^{max}=2$. The procedure consisted of (i) an extensive production of docking solutions for the ligand-receptor interaction, (ii) clustering of docking solutions into binding sites along the receptor structure and (iii) estimation of binding affinities using the free-energy perturbation (FEP) method. First completion of steps (i) through (iii) solved the ligand channel interaction for singly-occupied binding sites. Double occupancy of receptor sites were investigated by inputing the first generated ensemble of docked structures into another round of (i) through (iii) calculations. In detail, step (i) was accomplished by docking sevoflurane as a flexible ligand molecule against an MD-generated ensemble of membrane-equilibrated structures of the channel to properly handle the molecular flexibility of the protein receptor. Docking calculations were restricted to the pore domain region of the channel, free from the membrane surroundings. Step (ii) provided the location of δ*V*_*j*_ volumes lodging docking solutions for the ligand along the channel structure. Each of these volumes were treated as binding site regions in step (iii) calculations.

Following this procedure, binding constants were solved by inputting the FEP estimates into equations (11) and (12), allowing for direct solution of ${\rm{\rho }}({n}_{1},\ldots ,{n}_{s})$ via equation (7). Determination of ${\rm{\rho }}({\boldsymbol{R}})$ followed equations (13) and (14) with ρ(*n*_*j*_) calculated according to equation (15) and ${\rm{\rho }}({\boldsymbol{R}}|{n}_{j})$ estimated from the ensemble of docking solutions. Both estimates ${\rm{\rho }}({n}_{1},\ldots ,{n}_{s})$ and ${\rm{\rho }}({\boldsymbol{R}})$ were solved for sevoflurane concentrations in the range of 1–1000 mM. A detailed description of the calculations is provided as Supplementary Text.

## Results

Here, the main goal is to contribute a theoretical structure-based study of concentration-dependent binding of ligands against multiple saturable sites in membrane proteins. The work is illustrated in the context of binding of the general anesthetic sevoflurane to the well-understood open structure of the Kv1.2 channel^22, 23^. Our choice is justified as previous findings support that sevoflurane binds Kv1.2 through multiple sites^24^ to induce potentiation in a dose-dependent manner^25^.

### Ligand reservoir

In Theory and Methods, the equilibrium binding constant (see equation (6)) and following results are derived in the limit of a *homogeneous* diluted reservoir occupied by ligands at constant density $\bar{{\rm{\rho }}}$ and *excess* chemical potential $\bar{{\rm{\mu }}}$. Given that, we treated the system reservoir as a homogeneous aqueous solution despite its intrinsic inhomogeneity provided by the solvated lipid bilayer. An in-water *excess* potential of −0.1 kcal/mol (*cf*. Supplementary Information for details) was then estimated as the reservoir potential of sevoflurane and concentration effects were investigated for in-water densities $\bar{{\rm{\rho }}}$ in the range of 6.02 × 10^−7^ *Å* ^−3^– 6.02 × 10^−4^ *Å* ^−3^ (1 mM – 1000 mM in concentration units).

### Resolution of ligand sites on the protein receptor

**Figure 1:**
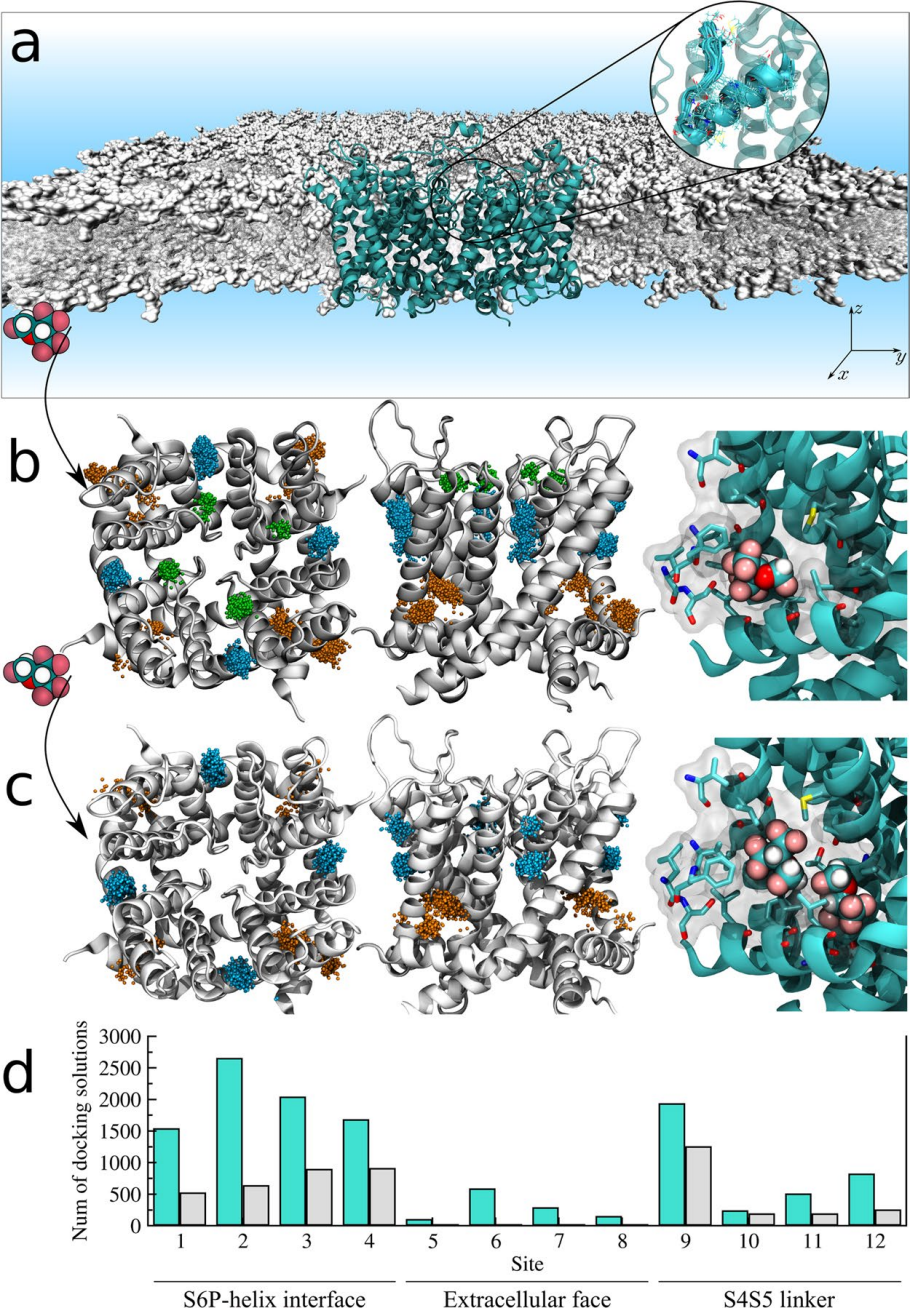
Resolution of sevoflurane sites on Kv1.2. (**a**) Molecular Dynamics simulation system containing the Kv1.2 channel (cyan) embedded in a fully-hydrated lipid bilayer (gray). Inset illustrates the MD-generated ensemble of channel structures considered for docking calculations. (**b**) Docking solutions for singly-occupied sites. Shown is the ensemble average structure of the channel along with the set of centroid configurations of sevoflurane (points) determined from docking. Centroid configurations of sevoflurane were clustered as a function of their location on the channel structure, that is at the S4S5 linker (orange), at the S6P-helix interface (blue) and at the extracellular face (green) next the selectivity filter. Each of these clusters was treated as an interaction site *j* for sevoflurane with volume δ*V*_*j*_. Inset shows a representative molecular structure resolved from docking. (**c**) Following another round of docking calculations started from structures in (**b**), solutions for doubly-occupied sites were resolved by determining if volumes δ*V*_*j*_ could accommodate the centroid positions of two docked ligands at once (inset). (**d**) Per site number of docking solutions for single (cyan) and double (gray) ligand occupancy. Voltage-sensor domains of the channel are not shown for clarity in (**b**,**c**).

From a total of ~15,000 docking solutions, clustering analysis returned 12 interaction sites for sevoflurane on Kv1.2 (Fig. 1). The interaction sites spread over the TM region of the channel at the S4S5 linker, at the S6P-helix interface of adjacent subunits and at the extracellular face. A minimum site-to-site distance of ~15 Å demonstrates their non-overlap distributions along the channel structure. Re-docking of sevoflurane generated in turn a total of ~5,000 solutions, solving the interaction of two ligands for all sites but the extracellular face.

### Ligand binding at low 1 mM concentration

From the docking ensemble, there are up to 3^12^ occupancy states of the channel that might contribute to sevoflurane binding. To evaluate this quantitatively, we performed a series of FEP calculations to estimate the per site binding affinity for one and two bound ligands via equation (12) (Table 1, Supplementary Fig. S1). As shown in Table 1, binding constants (or absolute binding free-energies) for the individual sites are heterogeneous and take place under a diverse range *ie*., 10^−7^–10^+7^ mM^−1^. There is however a clear decreasing trend of affinities involving sites respectively at the S4S5 linker, S6P-helix interface and extracellular face.

**Table 1:** FEP calculations and equilibrium binding constants for singly- and doubly-occupied sites^#^.

	Site	k_j_	${{\bf{W}}}_{{\bf{1}}}^{\ast }$ ± ε	K(0 … 1_j_ … 0)	ΔG°(0 … 1_j_ … 0)	k_j_	${{\bf{W}}}_{{\bf{2}}|{\bf{1}}}^{\ast }$ ± ε	${{\bf{W}}}_{{\bf{2}}}^{\ast }$	K(0 … 2_j_ … 0)	ΔG°(0 … 2_j_ … 0)
S6P-helix	1	0.053	−4.5 ± 0.2	5.93E−01	−3.8	0.196	−3.6 ± 0.2	−8.1 + −0.4	5.41E+00	−5.1
	2	0.141	−4.7 ± 0.2	1.92E−01	−3.1	0.137	−4.3 ± 0.2	−9.0 + −0.4	9.71E+00	−5.4
	3	0.084	−5.0 ± 0.2	6.86E−01	−3.9	0.084	−3.8 ± 0.3	−8.8 + −0.5	3.14E+01	−6.1
	4	0.036	−3.8 ± 0.3	3.26E−01	−3.4	0.174	−3.3 ± 0.4	−7.1 + −0.7	2.14E+00	−4.5
Ext. Face	5	0.530	1.5 ± 0.4	7.57E−07	4.3	—	—	—	—	—
	6	0.453	−0.8 ± 0.4	4.63E−05	1.8	—	—	—	—	—
	7	0.283	−0.8 ± 0.4	9.36E−05	1.4	—	—	—	—	—
	8	0.332	−4.2 ± 0.5	2.28E−02	−1.9	—	—	—	—	—
S4S5 linker	9	0.061	−6.4 ± 0.2	1.18E+01	−5.6	0.004	−4.0 ± 0.3	−10.4 + −0.5	7.45E+04	−10.7
	10	0.127	−7.5 ± 0.0	2.51E+01	−6.0	0.004	−5.2 ± 0.2	−12.7 + −0.2	1.18E+06	−12.4
	11	0.037	−6.7 ± 0.3	4.19E+01	−6.3	0.003	−7.0 ± 0.3	−13.7 + −0.6	4.97E+07	−14.6
	12	0.058	−5.6 ± 0.3	3.30E+00	−4.8	0.003	−6.1 ± 0.3	−11.7 + −0.6	1.00E+06	−12.3

^#^Units for force constants *k*_*j*_, ${W}_{1}^{\ast }\pm \epsilon$, $K(0\ldots {1}_{j}\ldots 0)$ and ${\rm{\Delta }}G^\circ (0\ldots {1}_{j}\ldots 0)$ are kcal/mol/Å^2^, kcal/mol, mM^-^^n^ and kcal/mol, respectively. Idem for doubly occupied sites. ${W}_{2}^{\ast }$ was computed as a two-step process ${W}_{2}^{\ast }={W}_{1}^{\ast }+{W}_{2|1}^{\ast }$ involving ligand coupling to a vacant site ${W}_{1}^{\ast }$ followed by binding of a second ligand at the preoccupied site ${W}_{2|1}^{\ast }$. FEP estimates and statistical errors (ε) were determined based on at least two independent FEP runs (*cf*. Supplementary Information for details).

**Figure 2:**
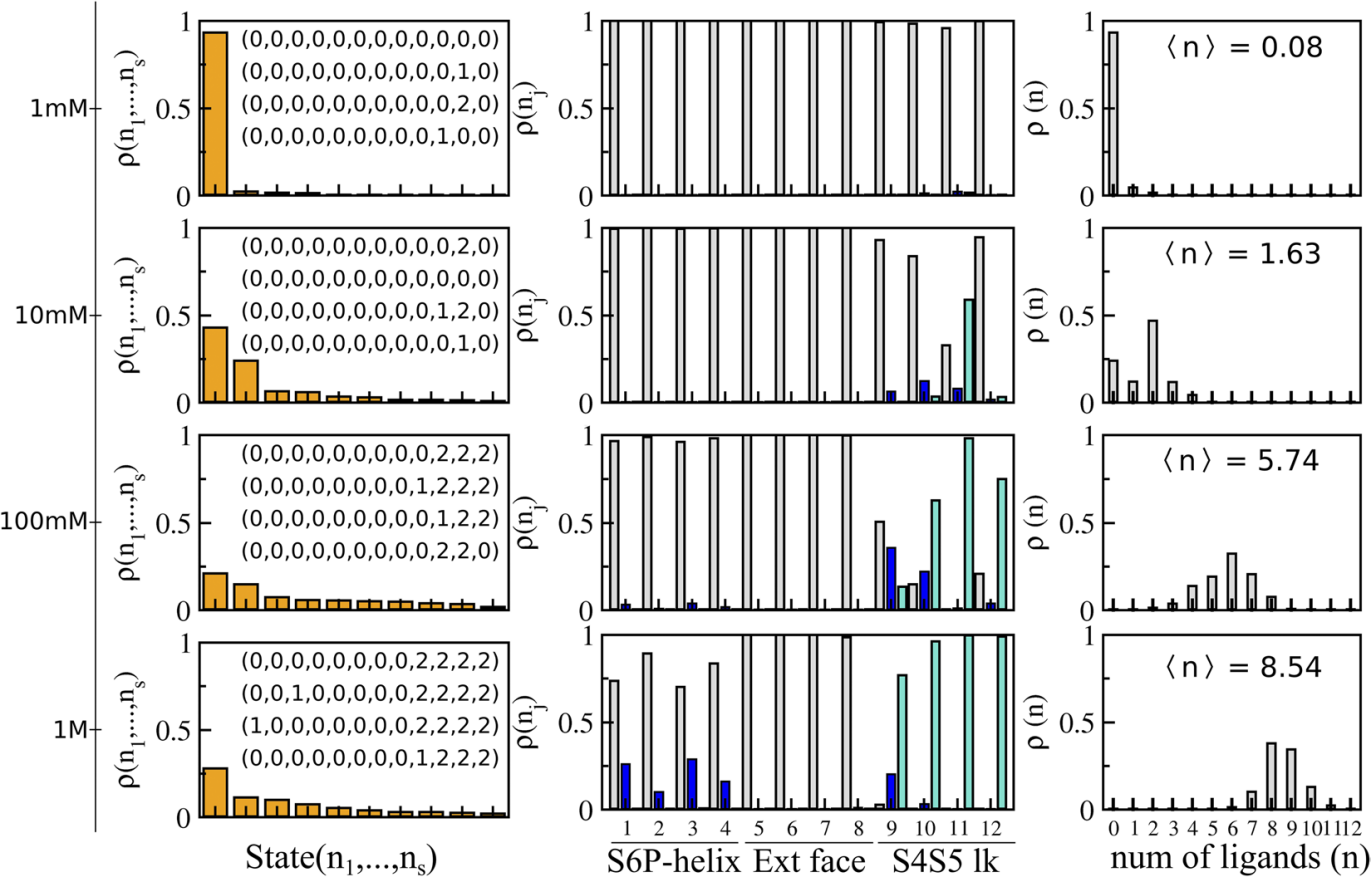
State-dependent binding probabilities for different concentrations of sevoflurane at the reservoir. (**a**) Sorted values of ${\rm{\rho }}({n}_{1},\ldots ,{n}_{s})$ over the channel occupancy states. Strings for the four most likely states are highlighted. (**b**) Marginal probabilities ρ(*n*_*j*_) of site *j*, for *n*_*j*_ = 0 (gray), *n*_*j*_ = 1 (blue) and *n*_*j*_ = 2 (cyan). (**c**) Probabilities ρ(*n*) for macrostates *O*(*n*). Here, ρ(*n*_*j*_) and ρ(*n*) were computed by coarse-graining over state probabilities in (**a**) according to equation (15) and Supplementary equation S1, respectively. Average number 〈*n*〉 of bound ligands as a function of the reservoir concentration are indicated in (**c**).

Under the assumption of independent sites, equilibrium constants ${\rm{{\rm K}}}({n}_{1},\ldots ,{n}_{s})$ for every occupancy state of the channel were then reconstructed from the per-site affinities (equation (11)) to determine the state probability ${\rm{\rho }}({n}_{1},\ldots ,{n}_{s})$ for a fixed ligand concentration in the reservoir (equation (7)). At low 1 mM concentration (Fig. 2), ${\rm{\rho }}({n}_{1},\ldots ,{n}_{s})$ is largely dominated by the probability of the empty state ${\rm{\rho }}({0}_{1},\ldots ,{0}_{s})$ implying only a small fraction of channel occupied states with non-negligible occurrences. Within this fraction, the most likely states involve single and double sevoflurane occupancy of the S4S5 linker as expected from the affinities reported in Table 1. Consistent with ${\rm{\rho }}({n}_{1},\ldots ,{n}_{s})$, the average number of bound ligands computed from equation (8) is 0.08 molecules.

**Figure 3:**
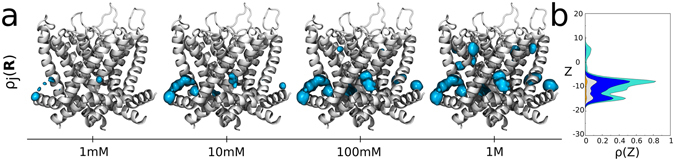
Position-dependent binding probabilities for different concentrations of sevoflurane at the reservoir. (**a**) Shown is the ensemble average structure of the channel along with the density ρ_*j*_(***R***) of sevoflurane (cyan) in each of the binding sites (isovalues of 9 × 10^−4^ Å^−3^). Voltage-sensor domains are not shown for clarity. (**b**) Projection of ρ_*j*_(***R***) along the transmembrane direction *z* of the system, ρ_*j*_(*z*). Note that the TM projection of ρ(***R***) across the entire channel-membrane system can be approximated by combining, into equation (16), the individual site projections ρ_*j*_(*z*) with projections elsewhere $\bar{{\rm{\rho }}}(z)=\bar{{\rm{\rho }}}\times A(z)$, where $\bar{{\rm{\rho }}}$ is the ligand reservoir density and *A*(*z*) is the membrane area.

The complex distribution of the multiple occupied states of the channel was readily visualized in three dimensions by mapping ${\rm{\rho }}({n}_{1},\ldots ,{n}_{s})$ into the position-dependent density ρ_*j*_(***R***) of sevoflurane in each of the binding sites. As prescribed in equation (14), this involved reweighing the marginal probability ρ(*n*_*j*_) of site *j* by the local equilibrium density of the ligand ${\rm{\rho }}({\boldsymbol{R}}|{n}_{j})$. Here, ρ(*n*_*j*_) was computed from equation (15) by coarse-graining over state probabilities in Fig. 2a whereas, ρ(***R***|*n*_*j*_) was calculated from the centroid distributions of docking solutions shown in Fig. 1b,c. As shown in Fig. 2, non-zero marginal probabilities for *n*_*j*_ = 1 and *n*_*j*_ = 2 take place only for sites at the S4S5 linker. The consequence for the distribution ρ_*j*_(***R***) at 1 mM is then clear, there is one dominant interaction spot for sevoflurane at the S4S5 linker that contrasts with vanishing densities at the other docking sites (Fig. 3). From equation (16), projection of ρ_*j*_(***R***) along the transmembrane direction *z* of the system, ρ_*j*_(*z*), stresses further the result.

### Concentration dependence of ligand binding and saturation effects

So far, our study supports that in average 0.08 sevoflurane molecules bind Kv1.2 at 1 mM, preferentially at the S4S5 linker. It is informative to clarify further the dependence of the results on concentration changes of the ligand in the reservoir. Figures 2 and 3 show ${\rm{\rho }}({n}_{1},\ldots ,{n}_{s})$, ρ_*j*_(***R***) and related quantities for reservoir concentrations of 10 mM, 100 mM and 1 M. Here, estimates at 1 M must be seen with caution as the presented formulation is designed to describe dilute conditions only. Expectedly, there is a clear shift of ${\rm{\rho }}({n}_{1},\ldots ,{n}_{s})$ towards states of the channel that enhances significantly the average number of bound ligands with concentration increase *ie*., ~1.63, 5.74 and 8.54 molecules. Careful inspection of ${\rm{\rho }}({n}_{1},\ldots ,{n}_{s})$ (or in a simpler way of ρ(*n*_*j*_)) confirms the major relevance of sites at the S4S5 linker over the entire concentration range, accompanied by an increasing importance of binding regions at the S6P-helix interface. In contrast, the probability density for sites at the selectivity filter remains negligible for all concentrations. The density of sevoflurane ρ_*j*_(***R***) and its TM projection ρ_*j*_(*z*) make sense of the results by showing the concentration dependent population of bound ligands.

As shown in Table 1, note for completeness that equilibrium constants for doubly-occupied sites are comparable to or even higher than estimates for one-bound ligand thus revealing important saturation effects in which one or two ligands can stably bind the channel at individual sites. The result is especially true for spots at the S4S5 linker.

### 4-fold symmetry

**Figure 4:**
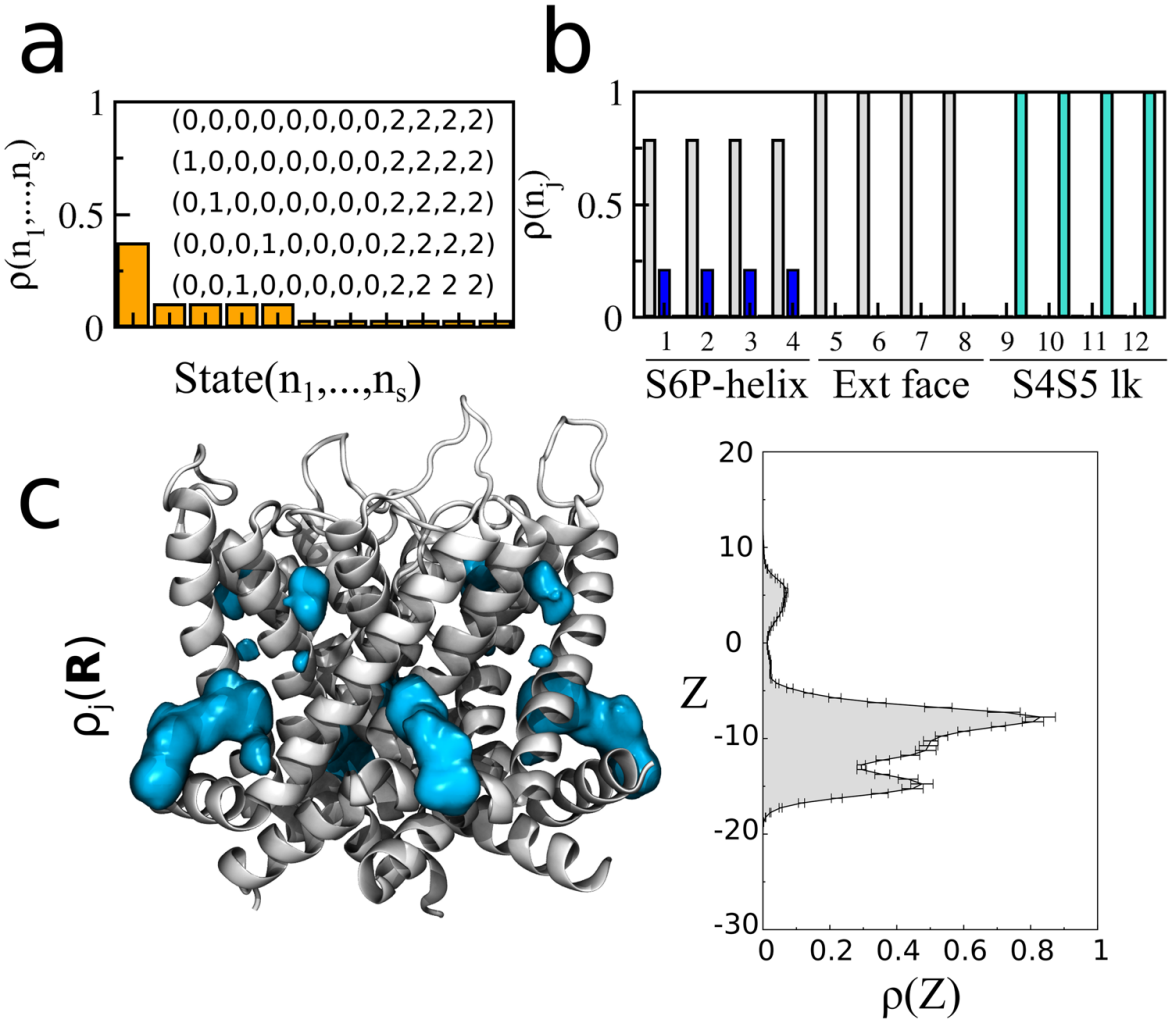
Symmetric state-dependent and position-dependent probabilities for sevoflurane at 1 M. (**a**) Sorted values of ${\rm{\rho }}({n}_{1},\ldots ,{n}_{s})$ over the channel occupancy states. Strings for the five most likely states are highlighted. (**b**) Marginal probabilities ρ(*n*_*j*_) of site *j*, for *n*_*j*_ = 0 (gray), *n*_*j*_ = 1 (blue) and *n*_*j*_ = 2 (cyan). (**c**) Shown is the ensemble average structure of the channel along with the density ρ_*j*_(***R***) of sevoflurane (cyan) in each of the binding sites (isovalues of 9 × 10^−4^ Å^−3^). Projection of ρ_*j*_(***R***) along the transmembrane direction *z* of the system ρ_*j*_(*z*) are also shown with error bars.

Kv1.2 is a homotetramer. Sevoflurane sites identified from docking are therefore indistinguishable across the channel subunits implying that there might effectively be 3 distinguishable regions for ligand binding on the channel structure that is, S4S5 linker, S6P-helix interface and selectivity filter. Table 2 shows average estimates and associated errors for sevoflurane affinities against each of these distinguishable regions. According to equation (18), statistical errors reflect the structural heterogeneity across the channel subunits implicit in the calculations as a result of finite MD-sampling of the Kv1.2 open conformation. Given that, Table 2 must provide us with statistically improved estimates when describing sevoflurane affinities to each of the distinguishable sites on Kv1.2. Following equation (17), we made symmetric all previous results for 1 M concentration of sevoflurane in the reservoir (Fig. 4). Reduction to symmetry causes redistribution of ligand-channel probabilities without modifying its average properties.

**Table 2:** Averaged-out estimates for singly- and doubly-occupied distinguishable sites^#^.

	K(0 …, 1_k_, … 0)	ΔG°(0 …, 1_k_, … 0)	K(0 …, 2_k_, … 0)	ΔG°(0 …, 2_k_, … 0)
S6P-helix	4.49E−01 ± 1.99E−01	−3.6	1.22E+01 ± 1.14E+01	−5.6
Ext. face	5.73E−03 ± 9.84E−03	−1.0		—
S4S5 linker	2.06E+01 ± 1.46E+01	−5.9	1.30E+07 ± 2.12E+07	−13.8

^#^Units for binding constants $K(0\ldots {1}_{k}\ldots 0)$ and absolute binding free-energies ${\rm{\Delta }}G^\circ (0\ldots {1}_{k}\ldots 0)$ considered for singly-occupied sites k are mM^−n^ and kcal/mol, respectively. Idem for doubly occupied sites.

## Discussion

Membrane proteins are primary targets for a large fraction of small molecule drugs that likely bind the protein receptor through complex concentration and saturation effects. Understanding the molecular structure of ligand binding thus prompts new advances in experimental and theoretical fronts, justifying the work herein. We presented a theoretical approach based on docking and FEP to study concentration-dependent interactions of ligands to multiple saturable sites in membrane receptors. Here, our study relies on two underlying assumptions that (i) docking can faithfully describe ligand interactions at protein sites and that (ii) binding events are independent over multiple sites. Specifically related to assumption (i), we have considered the generated ensemble of docking solutions to estimate the location of binding sites δ*V*_*j*_ and the local distribution of the ligand ρ(***R***|*n*_*j*_) in each of the identified sites. The generation of false positive hits is however a well documented drawback of docking algorithms as a result of limitations of the scoring function in describing ligand solvation energies and protein flexibility^26^. In this regard, the combination of extensive docking calculations against an ensemble of equilibrium receptor structures to handle protein flexibility and FEP calculations based on fine force-fields to accurately estimate solvation energies are critical aspects of the presented strategy to minimize such drawbacks^26^. Given the same limitations of the scoring function, it is also not guaranteed that all binding hits nor that ρ(***R***|*n*_*j*_) can be accurately known from docking. In this regard, although not considered here, it might be important to integrate docking results from different algorithms involving different scoring functions in order to characterize the bound ensemble. Still, thanks to the generality of the presented formulation, extension of the current approach to sampling techniques other than docking, including all-atom flooding-MD simulations^3, 6, 7, 11^, might also be an important refinement in that direction (*manuscript in preparation*). When compared to docking, flooding-MD applied to membrane protein has however the disadvantage of handling with full partition of the ligand into protein sites for which slow kinetics may reflect into high computational costs for sampling convergence. In relation with assumption (ii), it is true that for case specific systems, the additive PMF in equation (10) may be a severe approximation that will likely fail as soon as nearby sites are simultaneously occupied. Given that, elaboration of a proper treatment of site dependence in multiple binding events and evaluation of its usefulness will be highly welcome in future studies. Before that and for certain systems, the formulated work based on equation (10) must therefore be seen as an 0-level approximation of more elaborate and still more complex descriptions of the binding constant.

The approach is illustrated here in the context of sevoflurane binding to Kv1.2 over [1 mM − 1 M] and saturation conditions up to ${n}_{j}^{max}=2$. A detailed description of sevoflurane binding and its implications for Kv1.2 function exceeds the main scope of this contribution and will be published elsewhere. Still, we find it pertinent to discuss key results of the study. The model system was chosen as previous findings support that sevoflurane binds Kv1.2 through multiple sites^24^ to induce potentiation in a dose-dependent manner^25^. Specifically, sevoflurane shifts leftward the voltage-dependence of channel and increases its maximum conductance. Overall, our calculations demonstrate that sevoflurane binds Kv1.2 in a concentration dependent manner, binding preferentially the S4S5 linker and the S6P-helix interface over a range of concentrations. From a physical-chemical point of view, spots at these channel regions are primarily hydrophobic pockets (Supplementary Fig. S2) providing with favorable interaction sites for the uncharged sevoflurane molecule. In contrast to the aforesaid spots, sites nearby the selectivity filter of Kv1.2 are primarily hydrated amphiphilic pockets (Supplementary Fig. S2) that disfavors sevoflurane interaction as reflected in the free-energies shown in Table 1. The unfavorable binding free-energies for the singly-occupied site thus support that the non-negligible fraction of poses determined from docking (Fig. 1d) corresponds to low affinity or false positives.

Its is particularly worth of mention that our findings recapitulate independently very recent photolabeling experiments demonstrating that photoactive analogs of sevoflurane and propofol do interact at the S4S5 linker and the S6P-helix interface of Kv1.2 at the open-activated state^27, 28^. In detail, Leu317 and Thr384 were found to be protected from photoactive analogs, with the former being more protected than the latter. As highlighted in Supplementary Fig. S3, atomic distances of these amino-acid to bound sevoflurane molecules at the S4S5 linker and S6P-helix interface are found here to be respectively 5.5 ± 1.1 Å and 10.6 ± 1.1 Å, in average more or less standard deviation. Such intermolecular distances imply their direct interactions with bound sevoflurane in agreement with the measured protective reactions. Besides that, our calculations also recapitulate the stronger protection of Leu317 in the sense that, relative to sites at the S6P-helix interface, the affinity of sevoflurane is found here to be higher at S4S5 linker given its stable occupancy by one or two ligands. The result is also consistent with previous Ala/Val-scanning mutagenesis showing a significant impact of S4S5 mutations on the effect of general anesthetics on family members of K^+^ channels^5^. In special, a single residue (Gly329) at a critical pivot point between the S4S5 linker and the S5 segment underlines potentiation of Kv1.2 by sevoflurane^24^. When bound at the S4S5, sevoflurane is found here to be in proximity to that amino acid (Supplementary Fig. S3).

The stable interaction of sevoflurane at the S4S5 linker of Kv1.2 is also consistent with independent structure-based calculations showing binding of one or two sevoflurane molecules at the linker of the homologous bacterial sodium channel NaChBac^11^. On the other hand, the unfavorable or absent interactions at the central cavity and next the selectivity filter of Kv1.2 contrasts with sevoflurane binding at analogous regions of NaChBac^11^ due major structural differences between Na^+^ and K^+^ channels. Specifically, the central cavity of potassium channels misses open-fenestrations of the sodium relatives^29^ and K^+^-selective filters are sharply distinct from Na^+^-selective ones^30^. Because sevoflurane induces potentiation rather than blocking of Kv1.2, we read the negligible or absent density of the ligand at the central-cavity of the pore in Fig. 3 as a self-consistent result of the study.

Kv1.2 potentiation by sevoflurane has been attributed to stabilization of the open-conductive state of the channel^24^. Given the critical role of the S4S5 linker for the gating mechanism of the channel^22^, it is likely that sevoflurane-S4S5 interactions as found here are at the origins of the experimentally measured voltage-dependent component of anesthetic action. Besides that, it is also likely that binding of sevoflurane at the S6P-helix interface might interfere allosterically with the selectivity filter operation thus affecting channel’s conductance. Such hypotheses have been raised also in the context of anesthetic action on bacterial sodium channels^7, 11^. Corroboration of such hypotheses from a molecular perspective is however not trivial and will necessarily involve further structural studies to demonstrate how ligand binding affects protein equilibrium to modulate function.

As we advance in the early stages of the membrane structural biology field, our study treats and reveals a new layer of complexity in ligand binding that brings us novel paradigms to think the problem and to delineate research accordingly. Traditional methods have limited applicability to systems with multiple non-identical sites as such methods can only yield global- rather than relative-affinities for individual sites. Besides that, most of these methods provide us with apparent affinities derived from dose-response experiments which essentially are indirect measurements of the binding event. Ligand-induced modifications of the recorded ionic current of an ion channel is an example of such indirect measures. By gathering information at the level of individual sites that can be combined into the description of macrostates as well (see Supplementary equations S1 to S3), the approach here brings direct structural-level information that may therefore help to design and interpret experiments. For instance, affinity constants for individual sites can help rationalizing recordings from photoaffinity^8^ and NMR^14^ labeling measurements when probing ligand interactions to specific protein sites. Another important possibility may rely on the combination of our approach with measurements from high-resolution mass spectrometry^13^ and time-resolved x-ray/neutron interferometry^31^. Recent advances allow determining high-resolution spectra and electron-density profiles for membrane proteins in their native environment with concrete perspectives to determine such records for ligand-bound proteins as well. The link here between ${\rm{\rho }}({n}_{1},\ldots ,{n}_{s})$ and ρ_*j*_(***R***) can be therefore useful to resolve unique three-dimensional maps matching experiments. In this case, an auxiliary model distribution for the ligand heavy atoms would be required to describe the ligand electron density from the point distribution encoded in ρ_*j*_(***R***).

We believe the study is of broad interest by providing a common framework for investigation of ligands and membrane proteins, useful in producing new results in the field. To the best of our knowledge, Fig. 3 represents a deeper and first revealed structural view on the intricate mode of interactions that might take place between small ligands and membrane proteins. In particular, it becomes clear that from a molecular recognition standpoint, small ligands can be very promiscuous implying that not all binding events might elicit functional effects. Besides complex concentration and saturation effects, that promiscuous nature is also an important take home message that should guide new developments to properly account for ligand binding and its interplay with protein equilibrium and function.

## Electronic supplementary material

Supplementary information file
