# Supplementary material for: Concentration-Dependent Binding of Small Ligands to Multiple Saturable Sites in Membrane Proteins
Source: Sci Rep. 2017 Jul 18;7:5734. doi: 10.1038/s41598-017-05896-8 (PMC5516019; doi:10.1038/s41598-017-05896-8)
Supplement: Supplementary file 1 — Supplementary information file [file 41598_2017_5896_MOESM1_ESM.pdf]

Supplementary Information
Concentration-Dependent Binding of Small Ligands to Multiple Saturable Sites in Membrane Proteins

Letícia Stock, Juliana Hosoume and Werner Treptow*

Laboratório de Biologia Teórica e Computacional (LBTC), Universidade de Brasília DF, Brasil

SUPPLEMENTARY MATERIALS AND METHODS

Membrane Equilibrated Channel Structures. The Kv1.2 structure in the open state was obtained from Treptow and Tarek.[1] The construct was previously acquired via molecular dynamics (MD) simulations of the published x-ray crystal structure.[2] Modeling details and validation can be found in the original paper.

The channel structure was embedded in the lipid bilayer for MD relaxation and subsequent molecular docking of sevoflurane. Specifically, the structure was inserted in a fully hydrated and neutral (zwitterionic) all atom palmitoyloleylphosphatidylcholine (POPC) phospholipid bilayer. After assembled, the macromolecular system was simulated over an MD simulation spanning ~ 20 ns, at constant temperature (300 K) and pressure (1 atm), neutral pH, and with no applied TM electrostatic potential. The channel structure remained stable in its starting open conformation throughout the simulations. In the Kv1.2 simulation, the root mean-square deviation (rmsd) values for the whole TM domain, as well as for segments S5S6 (pore) and the S4S5 linker, range from 1.0 to 3.5 Å, which agrees with the structural drift quantified in previous simulations of the channel.

Molecular Docking. Using *AutoDock Vina,*[3] sevoflurane was docked against a MD-generated ensemble of 1200 structures of the channel. Docking solutions were resolved with an exhaustiveness parameter of 200, by searching a box volume of 50.0 x 50.0 x 100 $Å^3$ containing the pore domain region of the protein receptor (voltage sensors were excluded from docking). Sevoflurane was allowed to have flexible bonds for all calculations. Clustering of docking solutions was carried out following a maximum neighborhood approach.

Molecular Dynamics. All MD simulations were carried out using the program NAMD 2.9.[4] Langevin dynamics and Langevin piston methods were applied to keep the temperature (300 K) and the pressure (1 atm) of the system fixed. The equations of motion were integrated using a multiple time-step algorithm.[5] Short- and long-range forces were calculated every 1 and 2 time-steps respectively, with a time step of 2.0 fs. Also, periodic-boundary conditions were employed**.** Chemical bonds between hydrogen and heavy atoms were constrained to their equilibrium value. Long-range electrostatic forces were taken into account using the Particle Mesh Ewald (PME) approach.[6] The CHARMM36 force field[7] were applied and water molecules were described by the TIP3P model.[8] All the protein charged amino acids were simulated in their full-ionized state (pH=7.0). All MD simulations including FEP calculations (see next) were performed on local HPC facility at LBTC.

Free-Energy Perturbation (FEP). The *excess* chemical potential $\bar{\mu}$ associated with coupling of the ligand from gas phase to bulk water and $W^*_{n_j}$ associated with coupling of n_j ligands from gas phase to site j under restraints were quantified via FEP. Because computation of $\bar{\mu}$ does not depend upon the choice of concentration, so long as the same thermodynamic state is used for the solution and gas phases, we estimated the *excess* potential by considering one sevoflurane molecule embedded into a water box of 60 x 60 x 60 $Å^3$. $W^*_{n_j}$ was computed here by taking into considering the whole ligand-channel-membrane system.

All FEP calculations were performed in NAMD 2.9[4] by considering the Charmm-based parameters for sevoflurane as devised by Barber *et al.*[9] Starting from channel-membrane equilibrated systems containing bound sevoflurane as resolved from docking, forward transformation were carried out by varying the coupling parameter in steps of 0.05 (or for convergence purposes, in steps of 0.025 at final stages of the process). Each transformation then involved a total of 80 windows, each spanning over 32512 steps of simulation. For the purpose of improving statistics, free-energy estimates and associated statistical errors were determined using the simple overlap sampling (SOS) formula[10] based on at least two independent FEP runs.

Specifically for ligand-protein calculations, the free-energy change W^*_1 for singly-occupied sites was computed as a FEP process that involves ligand coupling to a vacant site. Differently, for doubly-occupied sites, W^*_2 was computed as a two-step FEP process involving ligand coupling to a vacant site W^*_1 followed by binding of a second ligand at the preoccupied site $W^*_{2|1}$. Because is a W^*_2 state function, the stepwise approach is equivalent to a single-step process involving simultaneous coupling of two ligands to the protein site that is, $W^*_2 = W^*_1 + W^*_{2|1}$. The colvars module[11] in NAMD 2.9 was used to apply the harmonic restraint potentials when computing these quantities. As described in the main text, the value of $W^*_{n_j}$ depends on the parameters of the restraint potential adopted in the FEP calculation *ie.*, the reference positions of the ligands in the bound state $\{\boldsymbol{R}^*_1, ..., \boldsymbol{R}^*_{n_j}\}$ and the magnitude of force constants $\{k_1, ..., k_{n_j}\}$. By minimizing the contribution of the restraint potential to the binding free-energy $W^*_{n_j}$, Roux and coworkers [12] devised optimum choices for the parameters

$$\{\boldsymbol{R}_1^* = \langle \boldsymbol{R}_1 \rangle, \ldots, \boldsymbol{R}_{n_j}^* = \langle \boldsymbol{R}_{n_j} \rangle\}$$

and

$$\{k_1 = \frac{3\beta^{-1}}{\langle \delta \boldsymbol{R}_1^2 \rangle}, \ldots, k_{n_j} = \frac{3\beta^{-1}}{\langle \delta \boldsymbol{R}_{n_j}^2 \rangle}\}$$

in which, $\langle \boldsymbol{R}_1 \rangle, \ldots, \langle \boldsymbol{R}_{n_j} \rangle$ and $\langle \delta \boldsymbol{R}_1^2 \rangle, \ldots, \langle \delta \boldsymbol{R}_{n_j}^2 \rangle$ are respectively the equilibrium average positions for each of the n_j bound ligands at site j and their corresponding mean-square fluctuations. Here, these parameters were estimated from the docking configuration space and the resulting force constants, in the range of 1.0 to 10.0 kcal/mol/Å^2, were considered for computations of the bound state.

Convergence of sampling. Here, a per-site measure for the ensemble of docking solutions effectively sampled in FEP was determined by quantifying the overlap $o(\boldsymbol{A}_j, \boldsymbol{B}_j)$ between the configuration space in both calculations[13]

$$o(\boldsymbol{A}_j, \boldsymbol{B}_j) = 1 - \frac{\sqrt{tr((\boldsymbol{A}_j^{1/2} - \boldsymbol{B}_j^{1/2})^2)}}{\sqrt{tr\,\boldsymbol{A}_j + tr\,\boldsymbol{B}_j}} \ ,$$

for $\boldsymbol{A}_j$ and $\boldsymbol{B}_j$ denoting covariance matrices associated respectively to FEP and docking samples at site j and, $\boldsymbol{A}_j^{1/2}$ and $\boldsymbol{B}_j^{1/2}$ their square roots. Specifically, $\boldsymbol{A}_j$ and $\boldsymbol{B}_j$ were determined as symmetric 3×3 covariance matrices for centroid positions $\boldsymbol{R}_j$ of the ligand at site j

$$\boldsymbol{X}_j = \langle (\boldsymbol{R}_j - \langle \boldsymbol{R}_j \rangle) . (\boldsymbol{R}_j - \langle \boldsymbol{R}_j \rangle)^T \rangle$$

and their square roots

$$\boldsymbol{X}^{1/2} = \boldsymbol{R}\, diag(\lambda_1^{1/2}, \lambda_2^{1/2}, \lambda_3^{1/2})\, \boldsymbol{R}^T$$

were solved from the column major eigenvectors $\{\boldsymbol{R}_1, \boldsymbol{R}_2, \boldsymbol{R}_3\}$ of the rotation matrix $\boldsymbol{R}$ and the associated eigenvalues $\{\lambda_1, \lambda_2, \lambda_3\}$. Note that the overlap equation $o(\boldsymbol{A}_j, \boldsymbol{B}_j)$ is expectedly 1 for identical samplings and 0 for orthogonal spaces.

Derivation of main text equation (5). Derivation of equation (5) is standard and follows from the coordinate transform $(\boldsymbol{r}^n) \rightarrow (\boldsymbol{R}^n, \boldsymbol{\Omega}^n, \boldsymbol{I}^n)$ involving the centroid positions $\boldsymbol{R}^n$, orientations $\boldsymbol{\Omega}^n$ and internal $\boldsymbol{I}^n$ degrees of freedom of the n ligands.

$$\int_{\delta V_1} d\boldsymbol{R}^{n_1} \ldots \int_{\delta V_s} d\boldsymbol{R}^{n_s} e^{-\beta W(\boldsymbol{R}^n)} =$$

$$\int_{\delta V_1} d\boldsymbol{R}^{n_1} \ldots \int_{\delta V_s} d\boldsymbol{R}^{n_s} \left[\frac{\int d\boldsymbol{r}_1 \delta[\boldsymbol{R}_1'(\boldsymbol{r}_1) - \boldsymbol{R}_1] \ldots \int d\boldsymbol{r}_n \delta[\boldsymbol{R}_n'(\boldsymbol{r}_n) - \boldsymbol{R}_n] \int_{V_{bulk}} d\boldsymbol{r}^{N-n} \int d\boldsymbol{r}^{M-N} e^{-\beta U(\boldsymbol{r}^M)}}{\int d\boldsymbol{r}^n \int_{V_{bulk}} d\boldsymbol{r}^{N-n} \int d\boldsymbol{r}^{M-N} e^{-\beta[U_o(\boldsymbol{r}^M) + \sum_{j=1}^{n} u^*(\boldsymbol{R}_j)]}} \times \frac{\int d\boldsymbol{r}^n \int_{V_{bulk}} d\boldsymbol{r}^{N-n} \int d\boldsymbol{r}^{M-N} e^{-\beta[U_o(\boldsymbol{r}^M) + \sum_{j=1}^{n} u^*(\boldsymbol{R}_j)]}}{\int d\boldsymbol{r}_1 \delta[\boldsymbol{R}_1'(\boldsymbol{r}_1) - \boldsymbol{R}_1^*] \ldots \int d\boldsymbol{r}_n \delta[\boldsymbol{R}_n'(\boldsymbol{r}_n) - \boldsymbol{R}_n^*] \int_{V_{bulk}} d\boldsymbol{r}^{N-n} \int d\boldsymbol{r}^{M-N} e^{-\beta U_o(\boldsymbol{r}^M)}} \right] =$$

$$\int_{\delta V_1} d\boldsymbol{R}^{n_1} \ldots \int_{\delta V_s} d\boldsymbol{R}^{n_s} \left[\frac{\int d\boldsymbol{r}_1 \delta[\boldsymbol{R}_1'(\boldsymbol{r}_1) - \boldsymbol{R}_1] \ldots \int d\boldsymbol{r}_n \delta[\boldsymbol{R}_n'(\boldsymbol{r}_n) - \boldsymbol{R}_n] \int_{V_{bulk}} d\boldsymbol{r}^{N-n} \int d\boldsymbol{r}^{M-N} e^{-\beta U(\boldsymbol{r}^M)}}{\int d\boldsymbol{r}^n \int_{V_{bulk}} d\boldsymbol{r}^{N-n} \int d\boldsymbol{r}^{M-N} e^{-\beta[U_o(\boldsymbol{r}^M) + \sum_{j=1}^{n} u^*(\boldsymbol{R}_j)]}} \times \frac{\int d\boldsymbol{r}_1 e^{-\beta u^*(\boldsymbol{R}_1)} \ldots \int d\boldsymbol{r}_n e^{-\beta u^*(\boldsymbol{R}_n)}}{\int d\boldsymbol{r}_1 \delta[\boldsymbol{R}_1'(\boldsymbol{r}_1) - \boldsymbol{R}_1^*] \ldots \int d\boldsymbol{r}_n \delta[\boldsymbol{R}_n'(\boldsymbol{r}_n) - \boldsymbol{R}_n^*]} \right] =$$

$$\int_{\delta V_1} d\boldsymbol{R}^{n_1} \ldots \int_{\delta V_s} d\boldsymbol{R}^{n_s} \left[\frac{\int d\boldsymbol{r}_1 \delta[\boldsymbol{R}_1'(\boldsymbol{r}_1) - \boldsymbol{R}_1] \ldots \int d\boldsymbol{r}_n \delta[\boldsymbol{R}_n'(\boldsymbol{r}_n) - \boldsymbol{R}_n] \int_{V_{bulk}} d\boldsymbol{r}^{N-n} \int d\boldsymbol{r}^{M-N} e^{-\beta U(\boldsymbol{r}^M)}}{\int d\boldsymbol{r}^n \int_{V_{bulk}} d\boldsymbol{r}^{N-n} \int d\boldsymbol{r}^{M-N} e^{-\beta[U_o(\boldsymbol{r}^M) + \sum_{j=1}^{n} u^*(\boldsymbol{R}_j)]}} \times \int d\boldsymbol{R}_1 e^{-\beta u^*(\boldsymbol{R}_1)} \ldots \int d\boldsymbol{R}_n e^{-\beta u^*(\boldsymbol{R}_n)} \right] =$$

$$[\prod_{i=1}^{n} (\frac{2\pi}{\beta k_i})^{\frac{3}{2}}] \times \int_{\delta V_1} d\boldsymbol{R}^{n_1} \ldots \int_{\delta V_s} d\boldsymbol{R}^{n_s} \left[\frac{\int d\boldsymbol{r}_1 \delta[\boldsymbol{R}_1'(\boldsymbol{r}_1) - \boldsymbol{R}_1] \ldots \int d\boldsymbol{r}_n \delta[\boldsymbol{R}_n'(\boldsymbol{r}_n) - \boldsymbol{R}_n] \int_{V_{bulk}} d\boldsymbol{r}^{N-n} \int d\boldsymbol{r}^{M-N} e^{-\beta U(\boldsymbol{r}^M)}}{\int d\boldsymbol{r}^n \int_{V_{bulk}} d\boldsymbol{r}^{N-n} \int d\boldsymbol{r}^{M-N} e^{-\beta[U_o(\boldsymbol{r}^M) + \sum_{j=1}^{n} u^*(\boldsymbol{R}_j)]}} \right] =$$

$$[\prod_{i=1}^{n} (\frac{2\pi}{\beta k_i})^{\frac{3}{2}}] e^{-\beta W_n^*}$$

In this case, the Jacobian of the transformation does not depend on the $\boldsymbol{R}^n$ degrees of freedom allowing for cancellation of $(\boldsymbol{\Omega}^n, \boldsymbol{I}^n)$ contributions. The effective volume $[\prod_{i=1}^{n} (\frac{2\pi}{\beta k_i})^{\frac{3}{2}}]$ thus results from the $3n$ -dimensional Gaussian integral appearing in line 4.

Derivation of main text equation (16). The spatial projection along the z direction of the system shown in equation (16) derives as

$$\begin{aligned}\rho(z) &= \int_{bulk} d\boldsymbol{R}\,\delta[z'(\boldsymbol{R})-z]\rho(\boldsymbol{R}) + \sum_{j=1}^{s}\left[\int_{\delta V_j} d\boldsymbol{R}\,\delta[z'(\boldsymbol{R})-z]\rho_j(\boldsymbol{R})\right] \\ &= \bar{\rho}\times A(z) + \sum_{j=1}^{s}\sum_{n_j=0}^{n_j^{max}} \rho(n_j)\int_{\delta V_j} d\boldsymbol{R}\,\delta[z'(\boldsymbol{R})-z]\rho_{n_j}(\boldsymbol{R}) \\ &= \bar{\rho}\times A(z) + \sum_{j=1}^{s}\rho_j(z)\end{aligned}$$

where, $A(z)=\Delta x \Delta y$ is the total area of the water-membrane region along the Cartesian x and y directions.

Coarse-graining over states $O(n_1,\ldots,n_s)$ **.** Description of the binding problem in terms of macrostates $O(n)$ can be of interest for macroscopic measurements. Consider any macrostate $O(n)$ of the system mapping an ensemble of accessible states $O(n_1,\ldots,n_s)$ in which n ligands bind the receptor regardless their specific distributions over the binding sites. Because $O(n)$ is degenerate, the probability density of the macrostate

$$\rho(n) = \sum_{n_1',\ldots,n_s'} \delta_{n',n}\,\rho(n_1',\ldots,n_s') \tag{S1}$$

can be determined by coarse-graining over the receptor states $O(n_1,\ldots,n_s)$ featuring exactly $n=n_1+\ldots+n_s$ bound ligands. Here, the Kronecker delta function $\delta_{n',n}$ ensures summation over states accessible to $O(n)$ only. The consequence is that the equilibrium constant $\mathrm{K}(n)$ for the process $O(0)+nL\Leftrightarrow O(n)$,

$$\mathrm{K}(n) = \sum_{n_1',\ldots,n_s'} \delta_{n',n}\,\mathrm{K}(n_1',\ldots,n_s') \tag{S2}$$

can be fully reconstructed from $\mathrm{K}(n_{1,}\ldots,n_s)$ constants. Supplementary equations (S1) and (S2) ensure the extension of the previous results to macrostate $O(n)$, where

$$\rho(n) = \frac{\bar{\rho}^{n}\mathrm{K}(n)}{\sum_{n'}\bar{\rho}^{n'}\mathrm{K}(n')} \tag{S3}$$

gives the probability density and

$$\Delta G^o(n) = -\beta^{-1}\ln[\mathrm{K}(n)\times(C^o)^n]$$

establishes the link between $\mathrm{K}(n)$ and the standard binding free-energy $\Delta G^o(n)$ associated to each of the states satisfying supplementary equation (S1).

1. Treptow, W. & Tarek, M. Environment of the gating charges in the Kv1.2 Shaker potassium channel. *Biophys. J.* **90,** L64-66 (2006).
2. Long, S. B., Campbell, E. B. & MacKinnon, R. Crystal structure of a mammalian voltage-dependentShakerfamily K+channel. *Science* **309,** 897–903 (2005).
3. Trott, O. & Olson, A. J. AutoDock Vina: Improving the speed and accuracy of docking with a new scoring function, efficient optimization, and multithreading. *J. Comput. Chem.* **31,** 455–461 (2010).
4. Phillips, J. C. *et al.* Scalable molecular dynamics with NAMD. *J. Comput. Chem.* **26,** 1781–1802 (2005).
5. Izaguirre, J. A., Reich, S. & Skeel, R. D. Longer time steps for molecular dynamics. *J. Chem. Phys.* **110,** 9853–9864 (1999).
6. Darden, T., York, D. & Pedersen, L. Particle mesh Ewald: An N·log(N) method for Ewald sums in large systems. *J. Chem. Phys.* **98,** 10089–10092 (1993).
7. Huang, J. & MacKerell, A. D. CHARMM36 all-atom additive protein force field: Validation based on comparison to NMR data. *J. Comput. Chem.* **34,** 2135–2145 (2013).
8. Jorgensen, W. L., Chandrasekhar, J., Madura, J. D., Impey, R. W. & Klein, M. L. Comparison of simple potential functions for simulating liquid water. *J. Chem. Phys.* **79,** 926–935 (1983).
9. Barber, A. F., Carnevale, V., Klein, M. L., Eckenhoff, R. G. & Covarrubias, M. Modulation of a voltage-gated Na+ channel by sevoflurane involves multiple sites and distinct mechanisms. *Proc. Natl. Acad. Sci.* **111,** 6726–6731 (2014).
10. Lu, N., Kofke, D. A. & Woolf, T. B. Improving the efficiency and reliability of free energy perturbation calculations using overlap sampling methods. *J. Comput. Chem.* **25,** 28–40 (2004).
11. Fiorin, G., Klein, M. L. & Hénin, J. Using collective variables to drive molecular dynamics simulations. *Mol. Phys.* **111,** 3345–3362 (2013).
12. Roux, B., Nina, M., Pomès, R. & Smith, J. C. Thermodynamic stability of water molecules in the bacteriorhodopsin proton channel: a molecular dynamics free energy perturbation study. *Biophys. J.* **71,** 670–681 (1996).
13. Hess, B. Convergence of sampling in protein simulations. *Phys. Rev. E* **65,** 031910 (2002).

SUPPLEMENTARY FIGURES

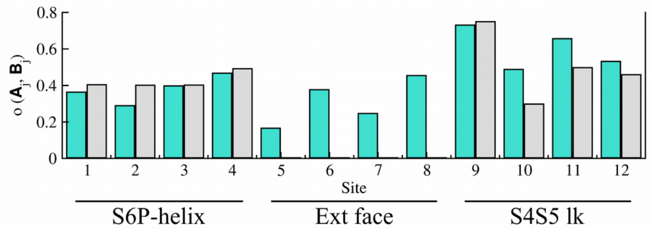

Supplementary Figure. S1. Configuration space overlap $o(\boldsymbol{A}_j, \boldsymbol{B}_j)$ between FEP and docking calculations (*cf.* Supplementary Materials and Methods). Note that overlap is larger than 0.25 for the majority of singly- (cyan) and doubly-occupied (gray) sites supporting that FEP samples successfully the volumes δV_j determined from docking.

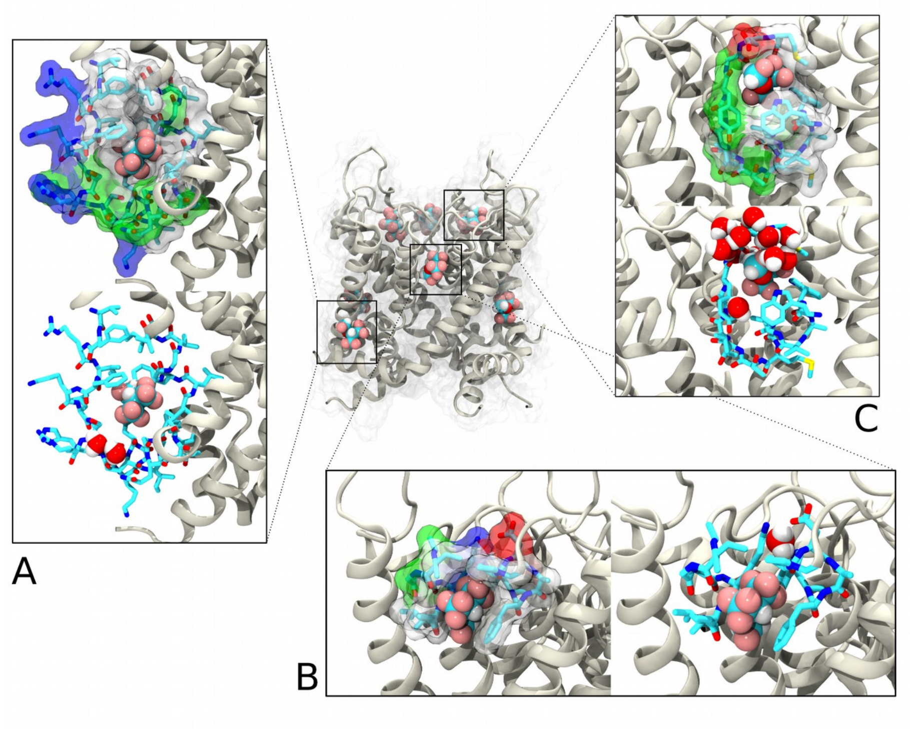

Supplementary Figure S2. Close view of sevoflurane interaction sites at Kv1.2 at the S4S5 linker (A), S6P-helix interface (B) and nearby the selectivity filter (C). The molecular surface in each of the binding sites is colored by physical-chemical properties of the constituting amino acids: hydrophobic (white), polar (green) and charged (blue or red). Note that sites (A) and (B) are primarily dehydrated hydrophobic pockets whereas the site nearby the selectivity filter is amphiphilic and largely hydrated.

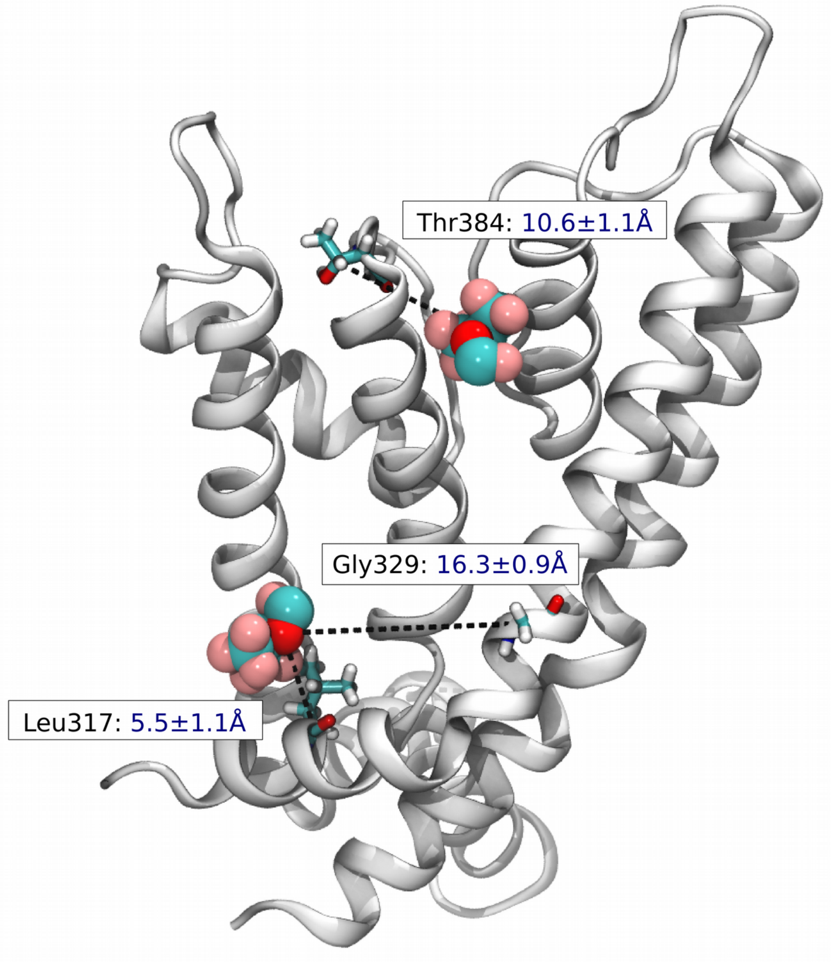

Supplementary Figure S3. Average atomic distances between the centroids of sevoflurane and photolabeled Kv1.2 residues. Distances were measured considering ensembles of both equilibrium protein structures inputed into docking searches, and sevoflurane docking poses pertaining to a given binding site. As highlighted in the image, average distance between sevoflurane geometric center when bound to S6P-helix site and Thr384 is 10.6±1.1Å; average distance of the ligand when bound to S4S5-linker site to residues Leu317 and G329 are respectively 5.5±1.1Å and 16.3±0.9Å.
